# Nuclear factor kappa B activation appears weaker in schizophrenia patients with high brain cytokines than in non-schizophrenic controls with high brain cytokines

**DOI:** 10.1186/s12974-020-01890-6

**Published:** 2020-07-17

**Authors:** Caitlin E. Murphy, Adam J. Lawther, Maree J. Webster, Makoto Asai, Yuji Kondo, Mitsuyuki Matsumoto, Adam K. Walker, Cynthia Shannon Weickert

**Affiliations:** 1 Schizophrenia Research Laboratory, Neuroscience Research Australia, Barker Street, Randwick, Sydney, NSW 2031 Australia; 2 School of Psychiatry, Faculty of Medicine, University of New South Wales, Sydney, NSW Australia; 3 Stanley Medical Research Institute, Kensington, Maryland USA; 4 Astellas Pharma Inc., Drug Discovery Research, Tsukuba, Japan; 5 Drug Discovery Biology Theme, Monash University, Parkville, Australia; 6 Department of Neuroscience & Physiology, Upstate Medical University, Syracuse, New York, USA

**Keywords:** Schizophrenia NF-κB inflammation cortex HIVEP2 Schnurri-2

## Abstract

**Background:**

High inflammation status despite an absence of known infection characterizes a subpopulation of people with schizophrenia who suffer from more severe cognitive deficits, less cortical grey matter, and worse neuropathology. Transcripts encoding factors upstream of nuclear factor kappa B (NF-κB), a major transcriptional activator for the synthesis of pro-inflammatory cytokines, are increased in the frontal cortex in schizophrenia compared to controls. However, the extent to which these changes are disease-specific, restricted to those with schizophrenia and high-neuroinflammatory status, or caused by loss of a key NF-κB inhibitor (HIVEP2) found in schizophrenia brain, has not been tested.

**Methods:**

Post-mortem prefrontal cortex samples were assessed in 141 human brains (69 controls and 72 schizophrenia) and 13 brains of wild-type mice and mice lacking HIVEP2 (6 wild-type, 7 knockout mice). Gene expression of pro-inflammatory cytokines and acute phase protein SERPINA3 was used to categorize high and low neuroinflammation biotype groups in human samples via cluster analysis. Expression of 18 canonical and non-canonical NF-κB pathway genes was assessed by qPCR in human and mouse tissue.

**Results:**

In humans, we found non-canonical upstream activators of NF-κB were generally elevated in individuals with neuroinflammation regardless of diagnosis, supporting NF-κB activation in both controls and people with schizophrenia when cytokine mRNAs are high. However, high neuroinflammation schizophrenia patients had weaker (or absent) transcriptional increases of several canonical upstream activators of NF-κB as compared to the high neuroinflammation controls. HIVEP2 mRNA reduction was specific to patients with schizophrenia who also had high neuroinflammatory status, and we also found decreases in NF-κB transcripts typically induced by activated microglia in mice lacking HIVEP2.

**Conclusions:**

Collectively, our results show that high cortical expression of pro-inflammatory cytokines and low cortical expression of HIVEP2 in a subset of people with schizophrenia is associated with a relatively weak NF-κB transcriptional signature compared to non-schizophrenic controls with high cytokine expression. We speculate that this comparatively milder NF-κB induction may reflect schizophrenia-specific suppression possibly related to HIVEP2 deficiency in the cortex.

## Introduction

The transcription factor nuclear factor kappa B (NF-κB) is a critical regulator of immune responses and controls the expression of various pro-inflammatory cytokines and acute phase proteins [1, 2] that are increased in the brain in people with schizophrenia [3–9]. Patients with elevated expression of these immune factors suffer from more severe symptoms [10–12], and fluctuations in the inflammatory milieu correlate with changes in symptom severity [13]. Thus, it is important to understand what factors are driving these inflammatory changes in people with schizophrenia. Recently, dysregulation of factors immediately upstream of NF-κB expression has been identified in the prefrontal cortex (PFC) of people with schizophrenia [14, 15]. Gene expression of members of 3 key processes are reportedly elevated in schizophrenia compared to controls: (1) receptors that initiate NF-κB signalling (interleukin-1 receptor type 1 (IL1R1), tumor necrosis factor receptor superfamily member 1A (TNFR1), toll-like receptor 4 (TLR4), cluster of differentiation 40 (CD40), lymphotoxin receptor beta (LTβR), and TNFR superfamily member 1B (TNFR2)); (2) subunits that form NF-κB dimers (proto-oncogene RelA (RelA), proto-oncogene c-Rel (cRel), NF-κB subunit 1 (NFKB1), and NF-κB subunit 2 (NFKB2)); and (3) kinases that induce their translocation into the nucleus (inhibitor of NF-κB kinase subunit alpha (IKKα), IKK beta (IKKβ), and NF-κB-inducing kinase (NIK)). Volk et al. also found increased expression of NF-κB inhibitor alpha (IκBα), which is upregulated by NF-κB activation itself in a negative feedback mechanism [16]. In contrast, another NF-κB inhibitor, human immunodeficiency virus type 1 enhancer binding protein 2 (HIVEP2) is decreased in schizophrenia cortex compared to controls [14]. These findings suggest that NF-κB is overactive in the cortex in people with schizophrenia and drives neuroinflammation in patients that may be causally relevant to their symptomatology.

**Fig. 1:**
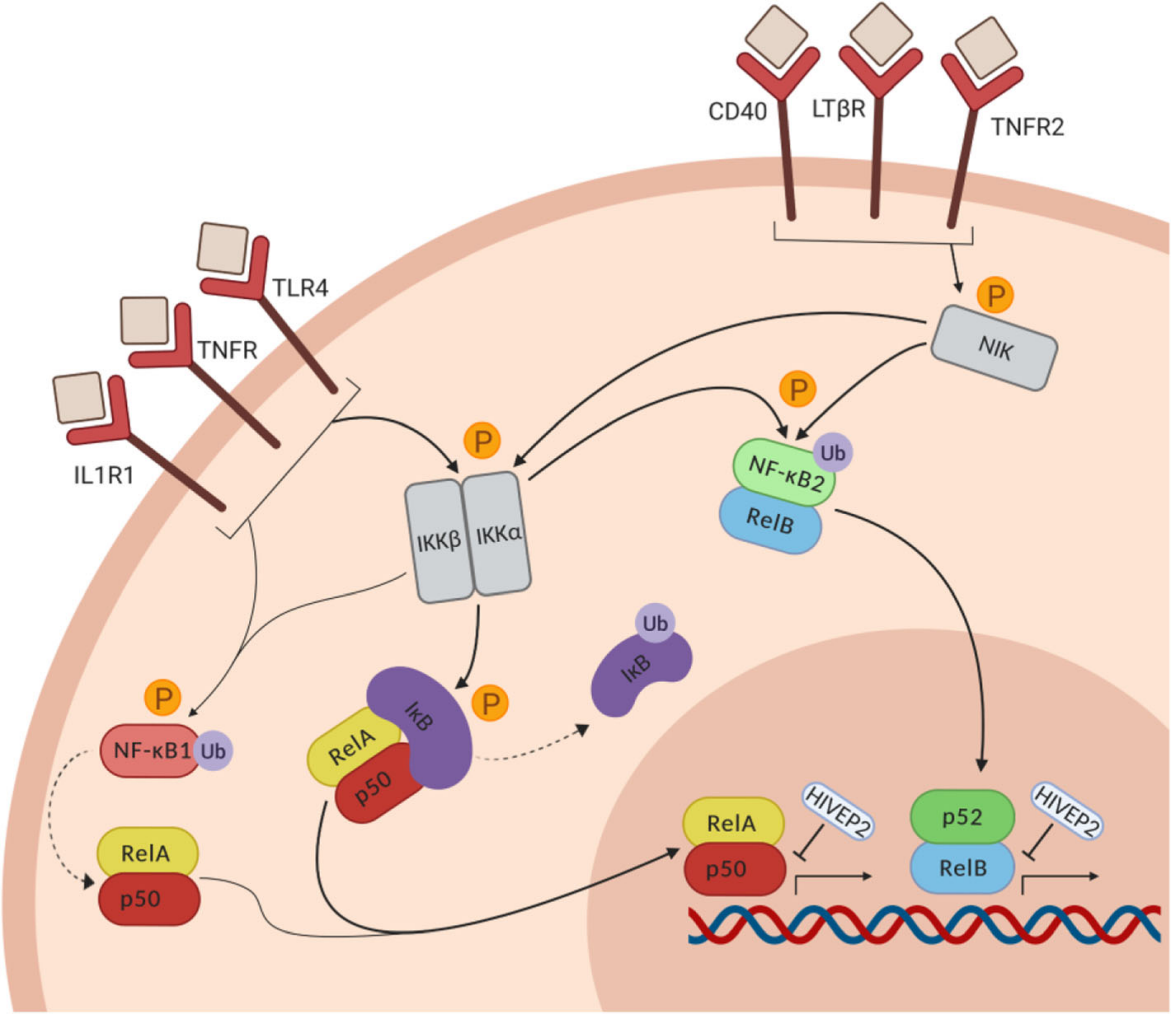
The NF-κB activation pathway. Receptors of the canonical arm of the pathway include IL1R1, TNFR, and TLR4. Activation of these receptors leads to the phosphorylation of both IKK and NF-κB1. The activated IKK complex tags IκB for proteasomal degradation, unmasking the nuclear localization sequence on RelA and allowing for nuclear localisation of the p50-RelA heterodimer. Activated IKKβ also phosphorylates NF-κB1, which is partially processed by the proteasome into mature subunit p50. p50 most commonly dimerizes with RelA, and the dimer either translocates to the nucleus or binds IκB and remains in the cytoplasm. Receptors of the non-canonical arm of the pathway include CD40, LTβR, and TNFR2. Activation of these receptors leads to the phosphorylation of NIK, which activates IKKα. Both NIK and IKKα are required for the phosphorylation of NF-κB2, which acts as an inhibitor while bound to RelB in the cytoplasm. Once tagged by NIK/IKKα, NF-κB2 undergoes partial processing by the proteasome into mature subunit p52. p52-RelB dimers then translocate to the nucleus. Rel-containing NF-κB dimers in the nucleus bind target gene DNA in a dimer- and cell type-specific manner to initiate transcription. HIVEP2 is a zinc finger protein that blocks NF-κB-induced gene transcription by binding κB sites on DNA

The NF-κB activation pathway consists of two arms—the canonical and non-canonical arms—that are induced by different receptors and involve different intracellular signalling proteins (Fig. 1). Both arms of the pathway require stimulus-induced activation of NF-κB-inducing kinases (NIK and IKKs) that tag NF-κB inhibitors for degradation (IκB, NF-κB1) or processing into mature subunits (NF-κB1/p50 and NF-κB2/p52). After this step, transcriptionally active NF-κB dimers translocate into the nucleus and bind κB sites on target DNA of cytokines to initiate gene transcription. Though there is a degree of overlap in target genes, κB binding preferences of dimers differ between the canonical and non-canonical arms ([17]; see Fig. 1). Additionally, the two arms have distinct functions in different cell types [1, 18]. Recent studies suggest overactivity of both arms of the NF-κB activation pathway in the cortex of people with schizophrenia [14, 15], but how this may differ from NF-κB activation in the normal human brain when inflamed has not been addressed.

Since degree of inflammation in schizophrenia relates to disease stage and symptom level [11, 12, 19, 20], it is likely that changes in cortical NF-κB activity occur only during active neuroinflammation and normalize (at least partially) during periods of symptom remission [12]. Thus, in the context of chronic schizophrenia, the subset of patients with neuroinflammation [3–6, 9, 11] plausibly drives the observed diagnostic effects in NF-κB gene expression. Furthermore, we hypothesize that the nature of neuroinflammation in schizophrenia may differ from that which occurs in non-schizophrenic controls who also have signs of neuroinflammation. Previous studies have only compared people with schizophrenia (who are more likely than people without schizophrenia to have inflammation) [3, 4] to non-schizophrenic controls; thus, it is possible that the differences observed are actually related to two underlying variables: diagnosis and neuroinflammatory status. Untangling these effects requires first identifying those cases that are in an active state of neuroinflammation based on increases in pro-inflammatory cytokines. Since previous studies have not considered the neuroinflammatory state of individuals, the extent to which changes in NF-κB-related transcripts are associated with inflammation or are specific to schizophrenia has not been explored. We sought to tease out which of these changes may be specific to, blunted or exaggerated in schizophrenia, and which may be related more generally to the neuroinflammatory state.

The driving factors behind cortical NF-κB dysregulation in schizophrenia are not known, and adult mice subjected to maternal immune activation in utero do not recapitulate changes in NF-κB-related transcripts [15]. HIVEP2, a protein that regulates NF-κB activity in the brain by preventing its target DNA-binding ([21, 22]; Fig. 1), is downregulated in the dorsolateral PFC of people with schizophrenia and cortical inflammation [14, 23]. HIVEP2 deletion in mice leads to several schizophrenia-like behavioral and neuropathological phenotypes [24] and allows us to test which NF-κB-related transcripts may be downstream of blunted HIVEP2 expression in schizophrenia or in controls. We measured the same 18 NF-κB-related messenger RNAs (mRNAs) in the cortex of mice lacking Schnurri-2 (Shn2; murine HIVEP2) and wild-type controls to determine the extent to which HIVEP2 deletion recapitulates schizophrenia-associated or neuroinflammation-associated NF-κB dysregulation within the mammalian brain. The primary aims of this study were to determine if inflammation-associated changes in upstream regulators of cortical NF-κB are different in people with schizophrenia than in non-schizophrenic controls, and to test the extent to which disease-associated changes in upstream regulators of cortical NF-κB would be consistent with those caused by a reduction in HIVEP2.

## Methods

### Human post-mortem brain tissue samples

Post-mortem dorsolateral PFC (Brodmann area 46) tissue was obtained from the New South Wales Tissue Resource Centre (TRC; 37 individuals with schizophrenia and 37 controls) and the Stanley Medical Research Institute (SMRI; 35 individuals with schizophrenia and 32 controls) (full demographics Supplementary Table 1). Details of brain collection and storage and determination of clinical and tissue factors for this cohort have been reported [4, 25]. Individuals were predetermined as having a low- or high-neuroinflammatory status or biotype based on expression levels of pro-inflammatory mRNAs as described in Fillman et al. [3] and Fillman et al. [4]. Briefly, the existence of inflammatory subgroups was tested in separate cohorts using a recursive two-step cluster analysis. The overall model quality was required to be > 0.5 (TRC) or > 0.4 (SMRI), with predictors removed if they did not contribute > 0.5 (TRC) or > 0.25 (SMRI) to the model on a scale of 0–1.0. Serpin Family A Member 3 (SERPINA3), IL-6, IL-8, and IL-1β transcripts were significant contributors to the model in both cohorts [3, 4]. TNF and IL1RL1 transcripts as well as brain pH also contributed to the formation of clusters in the SMRI cohort [4]. After combining cohorts, final group numbers were 57 low neuroinflammation controls, 12 high neuroinflammation controls, 42 low neuroinflammation schizophrenia, and 30 high neuroinflammation schizophrenia).

### RNA extraction, complementary DNA (cDNA) synthesis, and quantitative real-time PCR

For both cohorts, total RNA was extracted from the TRC dorsolateral PFC tissue using the Trizol method (Invitrogen, Carlsbad, CA, USA) per manufacturer protocol (Life Technologies). RNA quality and concentration were assessed on the Agilent Technologies 2100 Bioanalyzer and Nanodrop ND-1000 spectrophotometer. Complimentary DNA was synthesized from 1 μg total RNA per case using SuperScript®First-Strand Synthesis Kit IV and random hexamers following manufacturer instructions (Life Technologies). The mRNA expression of 18 NF-κB pathway genes was measured by reverse transcriptase-quantitative PCR (qPCR) in Fluidigm® BioMark™ HD system (South San Francisco, CA, USA) at the Ramaciotti Centre for Genomics (Kensington, NSW, Australia) using pre-designed Taqman Gene Expression Assays: IL1R1 (Hs00991010_m1), TNFR1 (Hs01042313_m1), TLR4 (Hs00152939_m1), CD40 (Hs00374176_m1), LTβR (Hs01101194_m1), TNFR2 (Hs00961750_m1), IKKα (Hs00989497_m1), IKKβ (Hs01559460_m1), NIK (Hs01089753_m1), IκBα (Hs00153283_m1), IκBβ (Hs00182115_m1), IκBε (Hs00234431_m1), RelA (Hs01042014_m1), cRel (Hs00968440_m1), RelB (Hs00232399_m1), NFKB1 (Hs00765730_m1), NFKB2 (Hs00174517_m1), and HIVEP2 (Hs00198801_m1). No reverse transcriptase controls and no template controls were included to rule out genomic DNA contamination and reagent contamination, respectively. Normalized relative quantities (2−ΔΔCt) of each mRNA were calculated using the geometric mean of three housekeeper genes that did not differ between diagnostic groups in either cohort (glyceraldehyde 3-phosphate dehydrogenase, Hs99999905_m1; TATA-binding protein, Hs00427620_m1; ubiquitin C, Hs00824723_m1).

### Schnurri-2 knockout mice

Seven Shn2 knockout (KO) mice and six wild-type control littermates were obtained by breeding heterozygotes with a C57BL/6J background and those with a BALB/cA background. Animals were group-housed under a 12-h light/dark cycle at a constant room temperature (23 ± 2 °C) and humidity (55 ± 15%), with free access to standard laboratory chow and water. At 17 weeks, mice were sacrificed via cervical dislocation and decapitation under isoflurane anesthesia. Whole brains were removed, and medial prefrontal cortex and infralimbic cortex samples were punched-out and snap frozen for RNA extraction and cDNA preparation (using total 1.5 μg RNA per mouse sample) as described above. The following probes were used for qPCR: IL1R1 (Mm00434237_m1), TNFR (Mm01182929_m1), TLR4 (Mm00445273_m1), CD40 (Mm00441891_m1), LTβR (Mm00440235_m1), TNFR2 (Mm00441889_m1), IKKα (Mm00432529_m1), IKKβ (Mm01222247_m1), NIK (Mm00444166_m1), IκBα (Mm00477798_m1), IκBβ (Mm00456853_m1), IκBε (Mm01269649_m1), RelA (Mm00501346_m1), cRel (Mm01239661_m1), RelB (Mm00485664_m1), NFKB1 (Mm00476361_m1), and NFKB2 (Mm00479807_m1). No reverse transcriptase controls and no template controls were included to rule out genomic DNA contamination and reagent contamination, respectively. Normalized relative quantities (2−ΔΔCt) of each mRNA were calculated using the geometric mean of two housekeeper genes (TBP, Hs99999905_m1; UBC, Hs00824723_m1).

### Statistical analyses

Independent samples *t* tests or independent samples Mann-Whitney *U* tests were used to confirm no differences in age at death, RIN, or PMI between controls and patients (Table 1) or between high and low inflammation biotypes (Table 2). Normalized relative mRNA quantities > 2 standard deviations from the group mean (low neuroinflammation control, high neuroinflammation control, low neuroinflammation schizophrenia, high neuroinflammation schizophrenia; wild-type mice, Shn2 KO mice) were considered outliers and excluded from analyses (average 3 low neuroinflammation control, 1 high neuroinflammation control, 2 low neuroinflammation schizophrenia, and 1 high neuroinflammation schizophrenia cases). mRNAs that were not normally distributed were log-transformed, square root-transformed, or inverted to achieve normality. The distribution of IκBβ mRNA did not normalize, so non-normal untransformed data was analyzed. Correlations were performed to assess the relationship between each mRNA and age at death, RNA integrity number (RIN), pH, and postmortem interval (PMI). If correlations were significant, the variable(s) was included as a covariate in subsequent analyses, excluding pH [26]. For schizophrenia samples, correlations were performed with each mRNA and calculated lifetime chlorpromazine equivalent to assess the relationship between expression of each transcript and anti-psychotic exposure. Two-way ANOVAs (diagnosis, inflammation, diagnosis × inflammation) were used to assess main effects of diagnosis and neuroinflammatory state and/or their interaction effect on expression of each gene. Planned post hoc *t* tests were used to compare gene expression between (1) low neuroinflammation controls and high neuroinflammation controls, (2) low neuroinflammation schizophrenia and high neuroinflammation schizophrenia, and (3) high neuroinflammation schizophrenia and high neuroinflammation controls. For mouse data, mean substitution for outliers (*n* = 1 for IκBα, *n* = 1 for TLR4, replacement with the group mean) was used to preserve statistical power. Independent samples *t* tests were used to examine differences in expression of the NF-κB-related mRNAs between Shn2 KO and wild-type mice.

**Table 1:** Comparison of demographic variables between diagnostic groups

	Control (N=69)	Schizophrenia (N=72)		
	Mean (SD)	Mean (SD)	t/U/χ² (df)	p-value
Age	48.01 (12.17)	47.07 (12.45)	t(139) = 0.46	0.65
Sex (m/f)	53/16	50/22	χ²(1) = 0.97	0.32
RIN	7.73 (0.78)	7.86 (0.83)	t(139) = -0.92	0.36
PMI (hrs)	27.15 (12.31)	29.89 (14.63)	U = 2743.00	0.26
pH	6.63 (0.28)	6.55 (0.28)	U = 1988.50	< 0.05

**Table 2:** Comparison of demographic variables between neuroinflammatory biotypes

	High (N=42)	Low (N=99)		
	Mean (SD)	Mean (SD)	t/U/χ² (df)	p-value
Age	47.33 (12.14)	47.62 (12.40)	t(139) = 0.13	0.90
Sex (m/f)	31/11	72/27	χ²(1) = 0.02	0.90
RIN	7.80 (0.83)	7.80 (0.80)	t(139) = -0.01	0.99
PMI (hrs)	27.52 (16.60)	28.98 (12.12)	U = 1852.00	0.31
pH	6.37 (0.28)	6.68 (0.23)	U = 825.50	< 0.01

## Results

### Membrane-bound NF-κB-activating receptor mRNAs are increased in inflammation

**Fig. 2:**
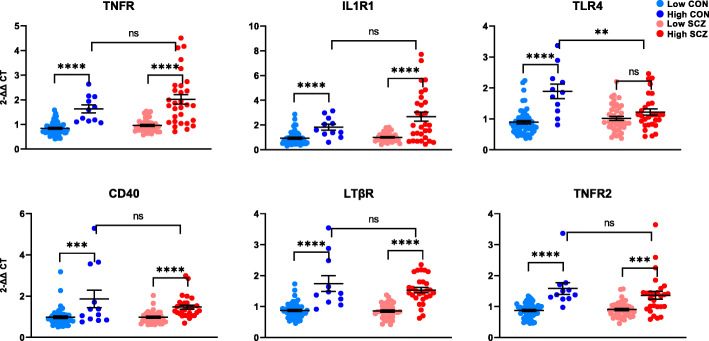
Gene expression of NF-κB-activating receptors in the post-mortem dorsolateral prefrontal cortex of controls (CON) and people with schizophrenia (SCZ). Diagnostic groups were stratified based on neuroinflammatory biotype at time of death (low neuroinflammation or high neuroinflammation). High neuroinflammation groups had higher expression of all receptors in both diagnoses, with the exception of TLR4 mRNA which did not differ between high neuroinflammation and low neuroinflammation in schizophrenia, and was decreased in patients with inflammation compared to non-schizophrenic controls with neuroinflammation. Diagnostically, TNFR mRNA was upregulated in schizophrenia compared to controls (*p* = 0.046). Error bars depict standard error of the mean. *p < 0.05, **p < 0.01, ***p < 0.001, ****p < 0.0001, ns = not significant

First, we detected viable expression levels of all 18 NF-κB-pathway members in human brain. We found a main effect of inflammation status for all six receptor mRNAs measured (IL1R1, TLR4, TNFR, TNFR2, LTβR, CD40; all *F* > 27.028, all *p* < 0.0001; Fig. 2). For TLR4, an interaction effect of diagnosis × inflammation was apparent such that the increase in mRNA related to high neuroinflammatory status was only evident in controls (*F*(1,131) = 11.995, *p* = 0.001). Compared to non-schizophrenic controls with neuroinflammation, patients with neuroinflammation had 35% less TLR4 mRNA (*t*(38) = 3.031, *p* = 0.004). In contrast, TNFR mRNA was upregulated in patients compared to controls overall (main effect of diagnosis *F*(1,130) = 4.061, *p* = 0.046, + 44%). We found no main effect of diagnosis on expression of IL1R1, TNFR2, LTβR or CD40 (all *F* < 1.559, all *p* > 0.214; Fig. 2). However, when we excluded the small number of high neuroinflammation controls (*n* = 12) from analysis, all six receptor mRNAs were increased diagnostically in schizophrenia compared to controls (all *p* < 0.006), consistent with previous findings.

### NF-κB-inducing kinase mRNAs are differentially upregulated in inflamed controls and inflamed patients

**Fig. 3:**
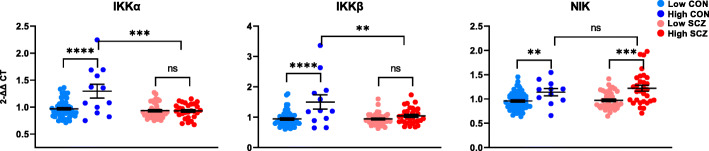
Gene expression of NF-κB-inducing kinases in the postmortem dorsolateral prefrontal cortex of controls (CON) and people with schizophrenia (SCZ). Diagnostic groups were stratified based on neuroinflammatory biotype at time of death (low neuroinflammation or high neuroinflammation). In controls, all three kinase transcripts (IKKα, IKKβ, NIK) were elevated in people with high neuroinflammation. In schizophrenia, only NIK was elevated in people with high neuroinflammation; expression of IKKα and IKKβ did not differ between neuroinflammatory biotypes. Diagnostically, IKKα mRNA was downregulated in schizophrenia compared to controls (*p* < 0.0001). Error bars depict standard error of the mean. **p < 0.01, ***p < 0.001, ****p < 0.0001, ns = not significant

Stimulus-dependent activation of NF-κB-inducing kinases occurs downstream of receptor-ligand binding and is necessary for signal propagation. In the canonical pathway, IKKα and IKKβ tag IκBs for degradation, which is essential for the translocation of NF-κB dimers into the nucleus to bind target DNA. We found a main effect of inflammation on IKKα and IKKβ transcripts (IKKα *F*(1,131) = 7.817, *p* = 0.006; IKKβ *F*(1,133) = 12.666, *p* = 0.001; Fig. 3), which were upregulated in high vs. low neuroinflammation groups. However, post hoc *t* tests revealed that this effect was only evident in controls (IKKα *t*(65) = 4.345, *p* < 0.0001; IKKβ *t*(65) = 4.299, *p* < 0.0001), and that high neuroinflammation patients had 28% and 30% lower expression of IKKα and IKKβ than high neuroinflammation controls, respectively (IKKα *t*(38) = 4.00, *p* < 0.001; IKKβ *t*(40) = 2.74, *p* < 0.01). We found a main effect of diagnosis on IKKα mRNA, in that levels were decreased in schizophrenia compared to controls (*F*(1,131) = 15.449, *p* < 0.0001), but when high neuroinflammation controls were excluded, there was no difference in expression diagnostically (*t*(121) = − 1.234, *p* = 0.220). For IKKβ, we were unable to detect a main effect of diagnosis with or without the inclusion of high neuroinflammation controls (all *p* > 0.147). The non-canonical pathway requires activation of NF-κB-inducing kinase NIK, which we found was increased in high neuroinflammation relative to low neuroinflammation individuals in both controls and patients (main effect of inflammation *F*(1,131) = 20.043, *p* < 0.0001; Fig. 3). We were unable to detect an effect of diagnosis on NIK mRNA unless high neuroinflammation controls were excluded, in which case we did find a main effect of diagnosis, where NIK mRNA was increased in schizophrenia (*t*(123) = − 2.652, *p* = 0.009).

### Increased expression of canonical NF-κB inhibitor mRNAs is associated with inflammation but is blunted in schizophrenia

**Fig. 4:**
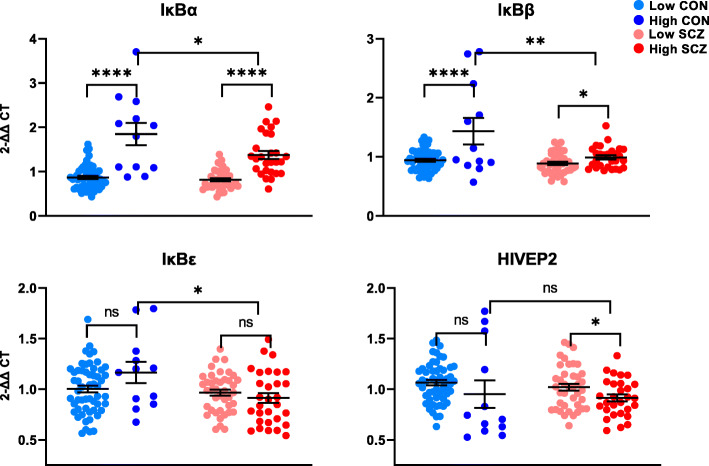
Gene expression of NF-κB inhibitors in the post-mortem dorsolateral prefrontal cortex of controls (CON) and people with schizophrenia (SCZ). Diagnostic groups were stratified based on neuroinflammatory biotype at time of death (low neuroinflammation or high neuroinflammation). Expression of IκBα and IκBβ was increased in high neuroinflammation groups compared to low neuroinflammation groups. However, both transcripts were higher in non-schizophrenic controls with neuroinflammation than in patients with neuroinflammation. Neuroinflammatory biotype was not associated with IκBε mRNA; however, high neuroinflammation controls had higher expression of IκBε than high neuroinflammation patients. All three traditional NF-κB inhibitors were decreased overall in schizophrenia compared to controls (all *p* < 0.034). HIVEP2 mRNA was associated with neuroinflammation in patients but not in controls and did not differ diagnostically. Error bars depict standard error of the mean. *p < 0.05, **p < 0.01, ****p < 0.0001, ns = not significant

IκBα, IκBβ, and IκBε remain largely in the cytoplasm bound to NF-κB dimers and are degraded in response to canonical IKK activation. The resynthesis of IκBs is stimulus-dependent and is driven by NF-κB in a negative feedback loop. As such, IκB mRNA upregulation could result from canonical or non-canonical NF-κB activation even though the latter occurs independently of IκB. We found a main effect of inflammation on IκBα and IκBβ transcripts in the human PFC, where IκBα and IκBβ were increased in high vs. low neuroinflammatory status (IκBα *F*(1,127) = 86.012, *p* < 0.0001, IκBβ *F*(1,137) = 11.671, *p* < 0.0001) to a greater extent in controls than in schizophrenia (Fig. 4). Because of this, both mRNAs were decreased in patients overall when testing the main effect of diagnosis (IκBα *F*(1,127) = 4.868, *p* = 0.029; IκBβ *F*(1,137) = 4.585, *p* = 0.034). When we excluded high neuroinflammation controls, IκBα mRNA was, in fact, higher in schizophrenia compared to controls overall (*F*(1,117) = 6.054, *p* = 0.015). IκBε mRNA was not associated with inflammation in either controls or patients, but was decreased in schizophrenia compared to controls overall (*F*(1,133) = 6.993, *p* = 0.009; Fig. 4). While we did not detect a main diagnostic difference in expression of HIVEP2, an atypical nuclear inhibitor of NF-κB, between schizophrenia and controls overall (*F*(1,130) = 0.001, *p* = 0.977), we saw an effect of inflammation within schizophrenia (*t*(67) = 2.151, *p* = 0.035; Fig. 4) where HIVEP2 was decreased in high neuroinflammation schizophrenia compared to low neuroinflammation schizophrenia. When omitting high neuroinflammation controls from analysis, HIVEP2 mRNA was decreased in schizophrenia compared to controls (*F*(1,120) = 7.822, *p* = 0.006) as expected.

### NF-κB subunit-encoding transcripts involved in canonical signalling—but not those involved in non-canonical signalling—are blunted in schizophrenia

**Fig. 5:**
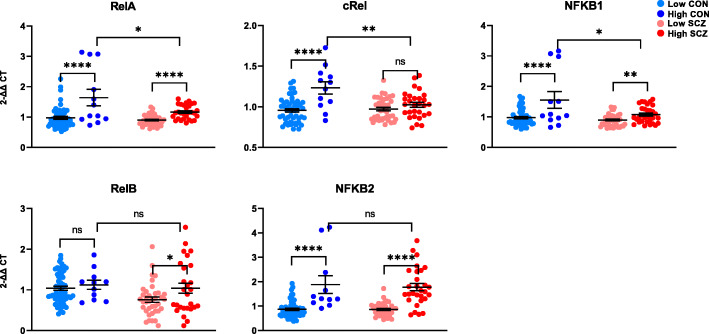
Gene expression of NF-κB subunits and subunit precursors in the post-mortem dorsolateral prefrontal cortex of controls (CON) and people with schizophrenia (SCZ). Diagnostic groups were stratified based on neuroinflammatory biotype at time of death (low neuroinflammation or high neuroinflammation). RelA, cRel, and NFKB1 mRNAs were increased in high neuroinflammation groups in both diagnoses; however, high neuroinflammation controls had significantly higher expression of all three transcripts compared to high neuroinflammation patients. RelB expression was associated with neuroinflammation only in patients and was decreased in schizophrenia compared to controls overall (*p* = 0.013). NFKB2 mRNA was upregulated in high neuroinflammation relative to low neuroinflammation and did not differ between non-schizophrenic controls with neuroinflammation and patients with neuroinflammation. Error bars depict standard error of the mean. *p < 0.05, **p < 0.01, ****p < 0.0001, ns = not significant

The most abundant transcriptionally active NF-κB dimer is comprised of RelA bound to p50 [27], which is constitutively processed from NF-κB1. The processing of NF-κB1 is enhanced by NF-κB receptor-ligand binding [28]. We found a main effect of inflammation on RelA and NFKB1 mRNA overall (RelA *F*(1,133) = 29.696, *p* < 0.0001; NFKB1 *F*(1,133) = 15.354, *p* < 0.0001; Fig. 5) and no main effect of diagnosis on either transcripts (RelA *p* = 0.374; NFKB1 *p* = 0.924). However, controls with cortical inflammation had greater expression of both RelA and NFKB1 transcripts than patients with cortical inflammation (RelA *t*(39) = 2.575, *p* = 0.014, + 29%; NFKB1 *t*(40) = 2.528, *p* = 0.016, + 31%). cRel, which also binds p50 to activate canonical NF-κB signalling [29], was associated with neuroinflammation via upregulation in controls but not in patients (interaction effect *F*(1,130) = 9.317, *p* = 0.003; Fig. 5). High neuroinflammation controls had 17% more cRel mRNA than high neuroinflammation patients (*t*(39) = 3.13, *p* = 0.003). Consequently, the main effect of diagnosis on cRel mRNA was a decrease in schizophrenia compared to controls overall (*F*(1,130) = 6.483, *p* = 0.012), but was not detected when high neuroinflammation controls were excluded (*F*(1,120) = 2.034, *p* = 0.156). NF-κB2 binds RelB and is processed into p52 to form the sole transcriptionally active NF-κB dimer of the non-canonical pathway [27]. In its unprocessed form, NF-κB2 can also inhibit canonical NF-κB signalling [30]. We found a main effect of inflammation on NFKB2 mRNA, where high neuroinflammatory status was associated with elevated expression regardless of diagnosis (*F*(1,127) = 71.314, *p* < 0.0001; Fig. 5), and found no difference in expression NFKB2 levels between controls with cortical inflammation and patients with cortical inflammation. We did not detect a main effect of diagnosis on NFKB2 expression overall (*F*(1,127) = 0.009, *p* =0.924). However, when we excluded high neuroinflammation controls, NFKB2 mRNA was upregulated in schizophrenia diagnostically (*F*(1,118) = 13.601, *p* < 0.0001). For RelB transcript, we found a main effect of diagnosis, where people with schizophrenia had decreased RelB mRNA overall, with (*F*(1,127) = 6.306, *p* = 0.013, − 17%) or without (*t*(118) = 2.496, *p* = 0.014, − 16%) the inclusion of high neuroinflammation controls (Fig. 5). Post hoc *t* tests also revealed that RelB was not changed in high neuroinflammation controls compared to low neuroinflammation controls; however, high neuroinflammation patients had greater RelB expression than low neuroinflammation patients (*t*(63) = 2.178, *p* = 0.033; Fig. 5).

### Expression of NF-κB-related mRNAs is related to brain pH

As expected, people with schizophrenia had significantly lower brain pH than controls (Table 1 [26]) and high neuroinflammation samples had significantly lower brain pH than low neuroinflammation samples (Table 2). Since higher inflammatory signaling is related to lower tissue pH in both controls and patients, almost all of the 18 NF-κB-related mRNAs were negatively correlated with brain pH (Supplementary Table 2). Thus, co-varying for pH would also remove some of the impact of diagnosis or inflammation status, and was not considered necessary as the groups were matched for RINs. People with schizophrenia were also significantly more likely than controls to be smokers at their time of death (Supplementary Table 1); however, 16 of 18 mRNAs measured did not differ between smokers and non-smokers (all *F* < 3.308, all *p* > 0.07). We did find that IL1R1 and TNFR transcripts were higher in the dorsolateral PFC of smokers (IL1R1 *F*(1,110) = 9.906, *p* = 0.002; TNFR *F*(1,111) = 5.376, *p* = 0.022).

### Anti-psychotic exposure is related to expression of some NF-κB-related mRNAs

Anti-psychotic drugs exert anti-inflammatory effects in the periphery by upregulating anti-inflammatory cytokines and reducing the expression of pro-inflammatory cytokines [31]. We ran correlations between each NF-κB mRNA and lifetime anti-psychotic exposure in patients to assess the relationship between anti-psychotics and NF-κB activation in the cortex. We found that the levels of three mRNAs TNFR, IκBα and NFKB2 were positively correlated with lifetime anti-psychotic (chlorpromazine equivalent) exposure (Supplementary Table 2).

### NF-κB transcripts are altered in the frontal cortex of Schnurri-2 knockout mice

**Fig. 6:**
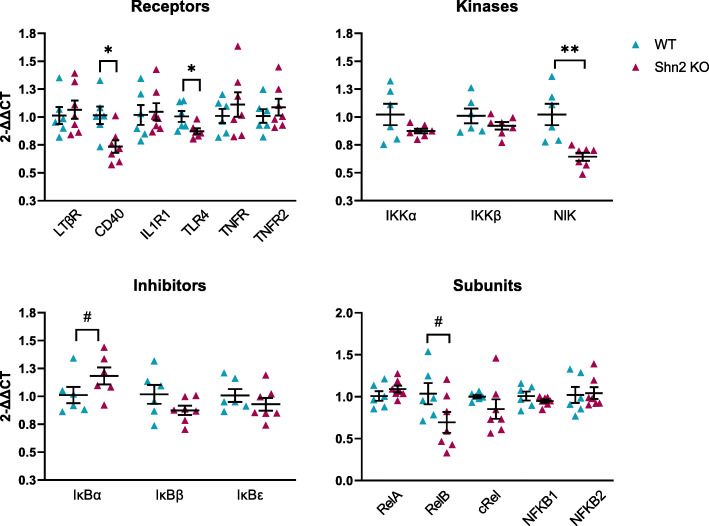
Gene expression of NF-κB transcripts in the prefrontal cortex of wild-type (WT) and Schnurri-2 knockout (Shn2 KO) mice. CD40, TLR4, and NIK mRNAs were significantly downregulated in Shn2 KO mice. Error bars depict standard error of the mean. *p < 0.05, **p < 0.01, #*p* = 0.08

HIVEP2 is decreased in the brain in high inflammation schizophrenia [15, 23], and HIVEP2 (Shn2) deletion induces neuroinflammation in adult mice [23, 24]. We compared the mRNA levels of factors upstream of NF-κB expression in the cortex of Shn2 KO mice to determine which of the changes seen in high neuroinflammation schizophrenia could be caused by HIVEP2 deficiency. In mice, Shn2 deletion led to substantial downregulation of NF-κB receptor mRNAs CD40 (− 28%; *t*(11) = 2.942, *p* = 0.013) and TLR4 (− 13%; *t*(11) = 2.424, *p* = 0.034; Fig. 6). The largest effect of Shn2 deletion in the prefrontal cortex was on NIK mRNA, which was reduced by 37% in Shn2 KO mice (*t*(11) = 3.932, *p* = 0.002; Fig. 6). Consistent with NF-κB activation, we found a trend of higher expression of IκBα (+ 17%) in Shn2 KO mice compared to wild-type mice (*t*(11) = − 1.930, *p* = 0.080). RelB mRNA was lower (− 33%) in Shn2 KO mice than wild-type mice but this effect also failed to reach significance (*t*(11) = 1.924, *p* = 0.081). Expression of many NF-κB transcripts (IL1R1, TNFR, TNFR2, LTβR, IκBβ, IκBε, IKKα, IKKβ, NFKB1, NFKB2, RelA, and cRel) did not differ between wild-type and Shn2 KO mice (all *p* > 0.13; Fig. 6).

## Discussion

In contrast to our expectation of exaggerated cortical immune activation in schizophrenia, we found evidence of blunted expression of canonical NF-κB members where schizophrenia and neuroinflammation co-occurred (compared to inflammation in the cortex of people without schizophrenia). Although individuals classified as having high cortical inflammation in both diagnostic groups showed widespread increases in NF-κB-related mRNAs, almost all (8 of 9 mRNAs) of the differences between the two inflamed groups were found in canonical NF-κB transcripts (TLR4, IKKβ, IκBα, IκBβ, IκBε, RelA, cRel, and NFKB1), which were decreased in high neuroinflammation schizophrenia compared to high neuroinflammation controls. TLR4 stimulation is a potent activator of microglia [32] and is implicated in both infectious and non-infectious (sterile) inflammation [33]. Furthermore, TLR4-mediated immune tolerance has been observed in microglia [34, 35], whereby chronic TLR4 stimulation results in reduced expression of TLR4 and microglial cytokine synthesis. Our finding of schizophrenia-specific relative suppression of TLR4 transcript plausibly reflects microglial quiescence in patients that is not seen in inflamed non-schizophrenic controls, and may reflect a more chronic neuroinflammatory environment in people with schizophrenia. Consistent with this hypothesis is the absence of clear cortical microglial activation in humans with schizophrenia in the postmortem brain [8, 36] and in living patients [37, 38]. A recent transcriptome-wide study in the post-mortem cortex of people with schizophrenia found that the microglial transcriptional network is actually downregulated compared to controls, despite an overall upregulation of NF-κB-associated genes [8]. This is in line with our findings and suggests that NF-κB may be suppressed in microglia but activated in other cells in the cortex of people with schizophrenia. However, mapping the changes in gene expression to specific brain cells capable of activating NF-κB and subsequent cytokine synthesis [39] will be necessary to confirm this possibility. The importance of canonical NF-κB signaling in cells of the myeloid lineage is highlighted by the impaired effector function of macrophages in the absence of cRel and NFKB1, two key subunits of this pathway [40]. In microglia, NFKB1/p50 is critical in shifting from the M1 pro-inflammatory phenotype into the M2 anti-inflammatory phenotype [41]. In this sense, canonical NF-κB in myeloid-derived cells is crucial for both effector function and the resolution of inflammation. While brain imaging studies have measured translocator protein (TSPO) in an attempt to assess microglial activation in schizophrenia, TSPO is not specific for microglia and cannot ascertain the role of microglia in the neuropathology of schizophrenia [42]. Interestingly, though deletion of nuclear NF-κB inhibitor HIVEP2 (Shn2) in mice leads to cortical immune activation [23, 24], we found significantly lower cortical mRNA for TLR4 in Shn2 KO mice compared to wild-type mice and concomitant lack of change in microglial activation marker Iba1 [23]. Further evidence of microglial suppression or lack of expected microglial activation in the context of HIVEP2 deficiency comes from our finding that the biggest NF-κB transcriptional change induced by Shn2 deletion was the downregulation of cortical NIK mRNA, which is highly enriched in microglia in mice [43] but not in human [5, 9]. It may be that lower HIVEP2 mRNA, which we found only in people with schizophrenia who are inflamed in the cortex, is directly responsible for the lack of TLR4 mRNA increase in this group and underpins the interaction effect of inflammation and diagnosis on TLR4 mRNA we observed here. Together, these findings point to reduced capacity to both respond to and resolve inflammation in the cortex of people with schizophrenia.

In another glial population involved in neuroinflammation, the astrocytes, the non-canonical NF-κB subunit RelB is crucial for the coordination of adaptive immunity; its upregulation in “tolerant” astrocytes is necessary for suppression of inflammatory signaling upon subsequent stimulation [44]. While we found no difference in expression of any of the NF-κB subunits (potential dimerization partners) in Shn2 KO mice, we did see a trend toward RelB mRNA reduction, suggesting Shn2-deficient astrocytes may be less able to appropriately suppress pro-inflammatory signaling. This may also be the case in people with schizophrenia, where we also found that RelB is decreased in the dorsolateral prefrontal cortex. An important limitation of our study that is inherent to homogenate-based assays is the inability to localize transcriptional changes to specific cell types. Indeed, in the absence of a foreign microbial antigen (“sterile” inflammation) as is the case in many cancers, in autoimmunity and possibly in schizophrenia, the neuroimmune environment is likely to reflect the efforts of several different cellular players searching for balance between inflammation and tolerance. The balance between these two states at any one time depends on many factors, including current stress levels, co-morbid physical conditions, chronicity of the inflammation, and frequency of flare-ups. Since some immune-related signaling molecules have beneficial effects in brain, such as maintaining and regulating synaptic function [45], a lower than normal level of cytokine inducers is not necessarily beneficial for normal human brain function. The comparatively weak NF-κB induction reported here could represent a failure of a primary inflammatory response or could be a consequence of chronic cytokine activation, either of which could be interpreted as pathological or protective [39]. Despite this ambiguity, we do know that neurons have minimal basal or inducible NF-κB [46], that reactive astrocytes are found in both Shn2 KO mice and high neuroinflammation schizophrenia [23, 24, 47], and that NF-κB is essential in the adoption of a pro-inflammatory phenotype in astrocytes [48, 49]. Additionally, since microglial signals determine the functional and phenotypic fate of reactive astrocytes [50], suppression of microglia might contribute to an inappropriately prolonged pro-inflammatory response from astrocytes. We thus speculate that reactive astrocytes contribute largely to the NF-κB transcriptional signature—and to pro-inflammatory cytokine expression—in neuroinflammation-associated schizophrenia in the putative absence of microglial activation.

One transcriptional change in Shn2 KO cortex that was opposite to what we found in high neuroinflammation schizophrenia cortex was downregulation of CD40 mRNA. Though its ligand CD40L is found mostly on T cells [51, 52] and this ligand is not normally detected in brain [5, 43, 52], CD40 upregulation is induced by pro-inflammatory cytokines in microglia and endothelial cells [53, 54]. As such, CD40 downregulation in Shn2 KO mice may represent another means by which to suppress microglial activation. Alternatively, since Shn2 KO mice have markedly less mature thymocytes than wild-type mice [55], we speculate that decreased endothelial expression of CD40 in Shn2 KO mice may also reflect a brain response to a reduction in circulating T cells. We found cortical upregulation of CD40 in patients with schizophrenia and elevated pro-inflammatory cytokine expression; however, this inflammation-associated increase was no different in non-schizophrenic controls, suggesting it is not a direct result of putative HIVEP2 deficiency (which was only seen in patients with neuroinflammation) in humans. Given other findings indicative of microglial suppression in schizophrenia, it is likely that cortical upregulation of the CD40 transcript in high neuroinflammation schizophrenia is driven by non-microglial cells that also express CD40 [5, 9].

While we found that exposure to anti-psychotic medication was positively correlated with expression of some NF-κB mRNAs, the magnitude of the correlations we report in this study is fairly low, and the effect of anti-psychotics on NF-κB activity in the brain may depend on the specific drug and inflammatory state. It has been reported that clozapine causes an increase in nuclear NF-κB activity in the mouse cortex [56], while haloperidol and olanzapine treatment do not appear to affect expression of NF-κB transcripts in monkey cortex [15]. Additionally, pre-treatment with risperidone inhibits LPS-induced increases in RelA in mice [57]. We found a positive correlation between lifetime anti-psychotic exposure and mRNA for NF-κB-activating receptor TNFR, NF-κB inhibitor IκBα, and NF-κB subunit precursor NFKB2 in the dorsolateral prefrontal cortex. IκBα and (unprocessed) NFKB2 are anti-inflammatory, so their correlation with anti-psychotic exposure may indicate anti-inflammatory effects of these drugs. However, these transcripts are also induced by NF-κB activation [58, 59]. Activation of the NF-κB pathway in murine glutamatergic neurons promotes dendritic spine and excitatory synapse formation [60, 61]; thus, it is possible that anti-psychotics activate NF-κB in these cells in humans and that this contributes to their therapeutic effect in treating schizophrenia. Alternatively, since high levels of cortical inflammation are associated with more severe symptomatology and, consequently, a higher anti-psychotic dose, it is plausible that greater NF-κB activation (perhaps specifically through TNFR, which was also associated with anti-psychotic exposure) leads to a requirement of more anti-psychotic treatment. Importantly, anti-psychotic agents can have both a direct anti-inflammatory effect and an indirect pro-inflammatory effect that is mediated by their association with weight gain and increased adiposity [62, 63], though the impact of BMI was not assessed in our study. Understanding how anti-psychotics may interact with the NF-κB pathway in both immune cells in the periphery and in brain cells are important aims for future studies.

## Conclusions

In sum, our findings show an association between cortical non-canonical NF-κB mRNA elevations and elevated neuroinflammatory status in a subset of people with schizophrenia that mainly appears to overlap with NF-κB mRNAs and elevated neuroinflammatory status in a subset of controls. Our results do find more specific diagnostic difference in transcriptional levels of canonical NF-κB pathway factors in those with high cortical inflammation suggesting they may utilize different cellular and intracellular means to achieve the same transcriptional ends (activation of cytokines). Further, our findings support that patients—but not non-schizophrenic controls—with cortical inflammation have decreased cortical HIVEP2 expression [14, 23], and that this may cause dysregulation of the NF-κB pathway in brain leading to putative blunting of normal inflammatory responses necessary to trigger various anti-inflammatory mechanisms.

## Supplementary information

**Additional file 1: Table S1**. Detailed cohort demographics. **Table S2**. Relationship of NF-κB transcripts in dorsolateral PFC with demographic variables

## Data Availability

The datasets generated and or analyzed during the current study are available in the SMRI repository, http://sncid.stanleyresearch.org.

## Abbreviations

NF-κB: Nuclear factor kappa B
PFC: Prefrontal cortex
IL1R1: Interleukin-1 receptor type 1
TNFR1: Tumor necrosis factor receptor superfamily member 1A
TLR4: Toll-like receptor 4
CD40: Cluster of differentiation 40
LTβR: Lymphotoxin beta receptor
TNFR2: Tumor necrosis factor receptor superfamily member 1B
RelA: Proto-oncogene RelA
cRel: Proto-oncogene c-Rel
NFKB1: Nuclear factor kappa B subunit 1
NFKB2: Nuclear factor kappa B subunit 2
IKKα: Inhibitor of nuclear factor kappa B kinase subunit alpha
IKKβ: Inhibitor of nuclear factor kappa B kinase subunit beta
NIK: Nuclear factor kappa B-inducing kinase
IκBα: Nuclear factor kappa B inhibitor alpha
HIVEP2: Human immunodeficiency virus type 1 enhancer binding protein 2
mRNA: Messenger RNA
Shn2 KO: Schnurri-2 knockout
TRC: Tissue Resource Centre
SMRI: Stanley Medical Research Institute
SERPINA3: Serpin family A member 3
qPCR: Quantitative PCR
