# Supplementary material for: Nuclear factor kappa B activation appears weaker in schizophrenia patients with high brain cytokines than in non-schizophrenic controls with high brain cytokines
Source: J Neuroinflammation. 2020 Jul 17;17:215. doi: 10.1186/s12974-020-01890-6 (PMC7368759; doi:10.1186/s12974-020-01890-6)
Supplement: Supplementary file 1 — Additional file 1: Table S1. Detailed cohort demographics. Table S2. Relationship of NF-κB transcripts in dorsolateral PFC with demographic variables [file 12974_2020_1890_MOESM1_ESM.docx]

**Supplementary Table 1. Detailed cohort demographics**

|  | **Control**  (N=69) | **Schizophrenia** (N=72) |  |  |
| --- | --- | --- | --- | --- |
|  |  |  | t/U/χ² (df) | p-value |
| Age | 48.01 (12.17) | 47.07 (12.45) | t(139) = 0.46 | 0.65 |
| Sex | 53 male/16 female | 50 male/22 female | χ²(1) = 0.97 | 0.32 |
| Race | 68 Caucasian/1 non-Caucasian | 70 Caucasian/2 non-Caucasian | χ²(1) = 0.30 | 0.58 |
| Hemisphere | 29 left/40 right | 37 left/35 right | χ²(1) = 1.24 | 0.27 |
| RIN | 7.73 (0.78) | 7.86 (0.83) | t(139) = -0.92 | 0.36 |
| PMI (hrs) | 27.15 (12.31) | 29.89 (14.63) | U = 2743.00 | 0.26 |
| pH | 6.63 (0.28) | 6.55 (0.28) | U = 1988.50 | **< 0.05** |
| Smoking around time of death | 18 yes/39 no/12 unknown | 46 yes/17 no/9 unknown | χ²(1) = 21.27 | **< 0.01** |
| Lifetime antipsychotics (chlorpromazine equiv., mg) | - | 3736500 (2500-32767875) | - | - |
| Duration of illness (years) | - | 24.54 | - |  |
| History of antidepressant use | - | 45 yes/27 no | - | - |
| Manner of death | 69 natural/0 suicide | 57 natural/15 suicide | χ²(1) = 16.09 | **< 0.01** |

Continuous variables presented as mean (SD) except lifetime antipsychotics which is presented as median (range). Bold p-values indicate significant difference between diagnostic groups.

**Supplementary Table 2. Relationship of NF-κB transcripts in dorsolateral PFC with demographic variables**

| **mRNA** | **Control** | | | | **Schizophrenia** | | | | |
| --- | --- | --- | --- | --- | --- | --- | --- | --- | --- |
|  | *Age* | *RIN* | *PMI* | *pH* | *Age* | *RIN* | *PMI* | *pH* | *CPZ equivalent* |
| *CD40* | -0.01 | -0.04 | **-0.35*** | **-0.50*** | 0.15 | 0.09 | -0.21 | **-0.34*** | 0.20 |
| *cREL* | **-0.28*** | 0.01 | -0.17 | -0.23 | 0.04 | -0.06 | 0.01 | 0.01 | 0.05 |
| *HIVEP2* | -0.23 | 0.10 | 0.10 | **0.26*** | -**0.33*** | 0.07 | 0.20 | **0.41*** | -0.23 |
| *IκBα* | -0.15 | -0.04 | -0.21 | **-0.50*** | 0.17 | -0.08 | **-0.25*** | **-0.63*** | **0.31*** |
| *IκBβ* | -0.06 | -0.01 | -0.93 | **-0.30*** | 0.10 | 0.03 | -0.10 | **-0.39*** | 0.12 |
| *IκBε* | **-0.41*** | 0.11 | 0.01 | 0.08 | 0.07 | -0.04 | -0.12 | 0.07 | 0.02 |
| *IKKα* | -0.07 | 0.02 | -0.22 | **-0.29*** | -0.53 | 0.15 | -0.03 | -0.09 | -0.06 |
| *IKKβ* | -0.15 | -0.08 | -0.19 | **-0.27*** | 0.05 | -0.08 | -0.18 | **-0.38*** | -0.07 |
| *IL1R1* | -0.17 | -0.12 | **-0.28*** | **-0.45*** | -0.03 | -0.11 | 0.04 | **-0.37*** | 0.12 |
| *LTβR* | -0.02 | -0.03 | -0.16 | **-0.54*** | 0.08 | -0.03 | -0.02 | **-0.61*** | 0.15 |
| *NIK* | **-0.27*** | 0.05 | -0.23 | **-0.28*** | 0.04 | -0.14 | 0.04 | **-0.29*** | 0.06 |
| *NFKB1* | -0.03 | -0.07 | -0.19 | **-0.34*** | 0.07 | 0.02 | -0.21 | **-0.45*** | 0.02 |
| *NFKB2* | -0.11 | 0.12 | **-0.29*** | **-0.37*** | 0.17 | -0.21 | -0.33 | **-0.56*** | **0.26*** |
| *RelA* | -0.02 | -0.06 | **-0.39*** | **-0.31*** | -0.02 | 0.13 | -0.20 | **-0.58*** | 0.07 |
| *RelB* | 0.07 | -0.07 | -0.01 | -0.09 | 0.07 | 0.05 | 0.18 | -0.18 | 0.22 |
| *TLR4* | -0.08 | 0.02 | **-0.35*** | **-0.52*** | 0.22 | -0.05 | -0.18 | **-0.47*** | 0.08 |
| *TNFR* | -0.21 | 0.08 | **-0.39*** | **-0.45*** | 0.14 | -0.14 | -0.07 | **-0.58*** | **0.26*** |
| *TNFR2* | **-0.27*** | 0.17 | -0.20 | -0.20 | -0.08 | 0.12 | -0.07 | **-0.35*** | 0.02 |

Values are correlation coefficients (r/ρ). Bold values with asterisk indicate significant correlations, p<0.05.
